# Communication at the Garden Fence – Context Dependent Vocalization in Female House Mice

**DOI:** 10.1371/journal.pone.0152255

**Published:** 2016-03-29

**Authors:** Svenja Hoier, Christine Pfeifle, Sophie von Merten, Miriam Linnenbrink

**Affiliations:** 1 Max Planck Institute for Evolutionary Biology, Plön, Germany; 2 Adam Mickiewicz University, Poznań, Poland; National Institute of Genetics, JAPAN

## Abstract

House mice (*Mus musculus*) live in social groups where they frequently interact with conspecifics, thus communication (*e*.*g*. chemical and/or auditory) is essential. It is commonly known that male and female mice produce complex vocalizations in the ultrasonic range (USV) that remind of high-pitched birdsong (so called mouse song) which is mainly used in social interactions. Earlier studies suggest that mice use their USVs for mate attraction and mate choice, but they could also be used as signal during hierarchy establishment and familiarization, or other communication purposes. In this study we elucidated the vocalization behaviour of interacting female mice over an extended period of time under semi-natural conditions. We asked, if the rate or structure of female vocalization differs between different social and non-social contexts. We found that female USV is mainly used in social contexts, driven by direct communication to an unknown individual, the rate of which is decreased over time by a familiarization process. In addition we could show that female mice use two distinct types of USVs, differing in their frequency, which they use differently depending on whether they directly or indirectly communicate with another female. This supports the notion that vocalization in mice is context dependent, driven by a reasonable and yet underestimated amount of complexity that also involves the interplay between different sensory signals, like chemical and auditory cues.

## Introduction

Communication signals can convey important information about certain environmental stimuli like food, nesting site, or a potential predator. They can however also code information about the signalling animal itself, about its reproductive or feeding status, as well as its social status and personality [1, 2, 3].

House mice (*Mus musculus*) form social groups with complex extended family structures, including multiple mating and inbreeding [4, 5, 6], but also pair bonding over extended periods of time exists [7]. Additionally, females frequently engage in communal nesting [8, 9, 10, 11]. In such complex social systems one would expect the evolution of a complex communication system [12]. A detailed description of the mouse social system can be found in a previous study [4]. Mice are known for their sophisticated olfaction, (*e*.*g*. [13]) they do however also produce two types of vocalizations, noisy squeaks in the human hearing range, and fine-structured ultrasonic vocalizations (USV) at frequencies above 30 kHz [14].

Although mouse USV has been studied for several decades, the exploration of functions of USV is still ongoing. Mice produce USVs during different social interactions. Mouse pups use USV as distress calls that elicit searching behaviour in the mother [15]. Adult male and female mice emit USVs that remind of high-pitched birdsong [16, 17]. The majority of studies on mouse USV work with male mice (*e*.*g*. [18, 19, 20, 21]). Male USV is important in the context of mate attraction and courtship [19, 20], and could serve as indicator for fitness, as well as family and population membership [19, 20, 21]. Only few studies exist on female vocalizations (*e*.*g*. [17, 22, 23, 24]), even though communal nesting is common in mice [8, 9, 10, 11] and represents an obvious reason for females to communicate with each other.

Studies with inbred mouse strains show that female USV is important for familiarization and hierarchy establishment in female-female encounters [22]. Females vocalize more in the presence of an unfamiliar as opposed to a familiar female, and the number of vocalizations is highest in the beginning of an encounter [22]. Also the feeding status of an intruding female, as well as oestrus state, pregnancy and age has been shown to influence the number of USVs recorded in female-female encounters [24, 25].

USV structure differs according to the type of interaction, *i*.*e*. same-sex or different sex [17], and is also specific to different non-social contexts [26]. Only recently it was found that male mice produce two distinct USV song types which they use differently, depending on whether they encounter a vivid female mouse or female urine [27], showing that mice can also produce different USV types within a certain social context, *i*.*e*. the actual presence of a mouse (directed communication) as opposed to the perceived former presence (undirected communication). Furthermore, this finding suggests that the functions of mouse USV should be evaluated on a finer contextual scale within one certain type of social interaction. Rats for example use three distinct types of 50 kHz vocalizations in the context of feeding, running, and fighting during same-sex interactions [28].

The aim of this study was to elucidate possible context specific mouse USVs during female-female encounters. We analysed female vocalizations in different social contexts (directed and undirected communication, as well as communication in the context of a male stimulus), and non-social contexts (*i*.*e*. food and nest) over three to four days. Furthermore, we studied the familiarization process of previously unfamiliar females over this relatively long period of time and ran repetitions to test whether we find the same pattern of USV usage in a second encounter between the same mice.

## Material and Methods

### Ethics statement

The animals used in this study are *Mus musculus*, a species that is not protected. At the time the progenitor animals were caught no permits for catching were necessary. When catching took place on the properties of private landowners, we got their oral permission to enter the property and catch mice. Catching took place in live traps, provided with food and shelter. Transportation to the laboratory, maintenance and handling were conducted in accordance with German animal welfare law (Tierschutzgesetz) and FELASA guidelines. Permits for keeping mice were obtained from the local veterinary office “Veterinäramt Kreis Plön” (permit number: 1401-144/PLÖ-004697). This study was specifically approved by the responsible animal welfare officer Prof. Gerhard Schultheiß. None of the animals used in the experiments were sacrificed during the study.

### Animals

The animals used in this study were progeny of wild *Mus musculus domesticus*, caught in the Cologne-Bonn region (Germany) in 2006, and kept for further breeding under outbreeding conditions to maintain natural genetic and behavioural variability.

In this study we tested 24 mature female mice from seven unrelated families. The animals were housed in groups together with their sisters (2 or 3 animals per cage). Sisters shared their home cage from the day of weaning onwards and were never separated before the experiment. Experimental pairs (later referred to as “recording partners”) were unrelated females from different cages. Animals from different cages never met directly, but they were housed in the same room within the same rack and could thus have smelled or heard individuals from other cages. As USV does not reach far and is additionally attenuated by cage walls and litter inside the cages, we do not expect any significant USV communication between the recording partners before the onset of experiments. Recording partners are thus considered unfamiliar.

At the time of the experiments they were between 9 and 12 months old, sexually unexperienced, and kept in the absence of any male mice for at least 3 months before the experiments.

**Fig 1:**
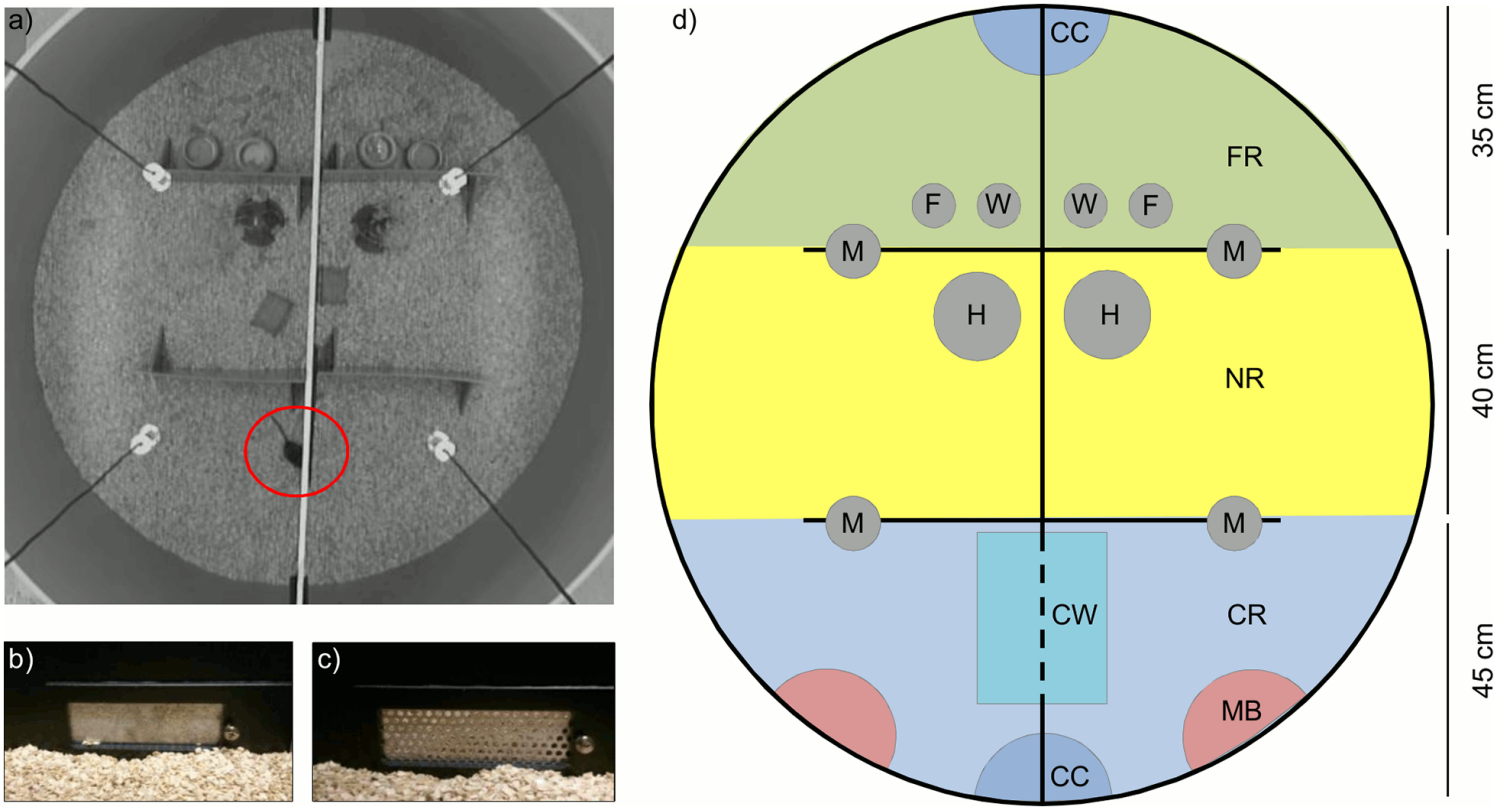
The experimental set-up. a) Photo of the arena set-up with one animal at the contact window (red circle). b) The contact window when closed and c) the open contact window. d) Schematic view of the arena with context regions as used for video scoring. The standard equipment for the experiments is colored in grey, the context regions that were used for video scoring are marked in different colors and inscribed with following abbreviations: CC = contact corners (dark blue); CR = contact region (light blue); CW = contact window (turquoise); F = food; FR = food region (green); H = red Plexiglas house; M = microphone, hanging from the top (see a); MB = male bedding (red); NR = neutral region (yellow); W = water.

Before and after the individual experiments all mice were housed under standard lab conditions with a dark-light cycle of 12 h, with lights on and off at 7 am/pm, temperature 16–24°C and 24–81% air humidity. Cages were equipped with bedding (Rettenmaier, Germany), nesting material and enrichment, food and water were provided *ad libitum*. During the experiment, mice lived under semi-natural conditions (Fig 1) provided as well with bedding, nesting material, enrichment, and food and water *ad libitum*.

### Experimental design

We aimed at examining the context specific vocalization of female mice. For this, we recorded the vocalizations and behaviour of wild female mice in a semi-natural enclosure environment (arena) over three to four consecutive nights. The arena was circular and built from grey PVC with a diameter of 120 cm and 73 cm height (Fig 1a). It was separated into two equally sized compartments by a black PVC-wall. Two unfamiliar female mice were placed in the arena, one in each compartment. The two compartments were connected via a small cavity in the separation wall (16 cm wide, 5.5 cm high; Fig 1b and 1c). Different metal plates could be inserted into this cavity to either close it completely using a solid metal plate (Fig 1b), or allowing contact between recording partners using a perforated metal plate as a contact window (holes measured 5 mm, with 3 mm spacing in-between, Fig 1c).

Small walls made of grey PVC (20 cm high, 35 cm long) were used to physically separate three context regions within each compartment that corresponded to certain social or environmental stimuli: (1) food region (FR), with food and water provided *ad libitum*; (2) nest region (NR), with a Plexiglas house and paper stripes for nesting; and (3) potential contact region (CR), the region where the window within the separation-wall allowed chemical, auditory and partly physical contact, when opened (Fig 1d). Walls between different compartments allowed an unambiguous scoring of the location of an animal within the compartments, and ensured that direct communication via auditory and visual signals was only possible when both animals were within the contact region of their respective compartment.

### Experimental procedure

We tested 12 female pairs and each experiment was started by introducing two females from different cages into the arena, one in each compartment, at 4 p.m. The females were then allowed to accommodate to their new environment for one night, without having direct contact with their recording partner in the other compartment, *i*.*e*. the contact window was closed (Fig 1b). It is likely that the recording partners sensed the mouse in the other compartment via smell already in this first night. After 24h of accommodation we exchanged the full metal plate in the contact window by a perforated one (Fig 1c), now allowing direct social contact between recording partners.

From here on experiments were subdivided into two subsets, A and B. Group A was exposed to a mixture of bedding from three adult male mice (presented in the contact region, Fig 1d) in the 3^rd^ night of the experiments. The contact window was kept open. Group B ran undisturbed for another night (night 3) with the contact window left open. The male bedding was provided in an additional 4^th^ night. For both experimental groups audio and video recordings were started directly after the mice were introduced into the arena and ran undisturbed until the experiment was finished after three or four nights, respectively.

By contrasting Group A and Group B we were able to disentangle the effects of introducing a male—and thus possible mating partner—stimulus and a potential familiarity effect between the females. Additionally, five recording pairs (MS_03, MS_04, MS_05, MS_06, MS_10; Table 1) were exposed to a second experimental trial, according to the procedure of Group A, four months after their first trial in order to check whether individual pairs were consistent in the quantity of emitted USVs.

**Table 1:** Number of songs per recording night and context region. Given is the number of songs per context region in each recording night and the number of songs in each recording night (sum), separately for each pair of females and summed over all experiments (sum all). The number of songs per night for the five repetitions is given in brackets. Context regions are: FR = food region, NR = nest region, CC = contact corners, CR = contact region.

	night 1					night 2					night 3					night 4				
Pair	sum	FR	NR	CC	CR	sum	FR	NR	CC	CR	sum	FR	NR	CC	CR	sum	FR	NR	CC	CR
MS_01	**33**	11	3	9	10	**179**	6	16	22	135	**80**	7	9	23	41	**0**	-	-	-	-
MS_02^(1)^	**26**	3	12	6	5	**113**	2	7	24	80	**0**	-	-	-	-	**0**	-	-	-	-
MS_03	**11 (7)**	5	3	0	3	**14 (39)**	0	1	1	12	**12 (21)**	0	0	0	12	**0**	-	-	-	-
MS_04	**26 (22)**	3	2	17	4	**27 (39)**	6	2	3	16	**31 (25)**	1	1	20	9	**0**	-	-	-	-
MS_05	**24 (19)**	5	6	7	6	**364 (413)**	7	24	35	298	**226 (166)**	13	10	24	179	**0**	-	-	-	-
MS_06	**48 (48)**	7	3	33	5	**130 (173)**	5	18	14	93	**56 (73)**	1	6	5	44	**0**	-	-	-	-
MS_07^(2)^	**24**	5	3	11	5	**117**	7	5	20	85	**28**	0	0	15	13	**31**	0	2	23	6
MS_08	**34**	2	3	28	1	**72**	1	2	21	48	**43**	0	0	23	20	**49**	2	4	20	23
MS_09	**7**	1	4	0	2	**13**	0	0	3	10	**5**	0	1	1	3	**11**	0	4	4	3
MS_10^(2)^	**35 (39)**	3	6	22	4	**269 (152)**	1	6	28	234	**92 (107)**	1	5	23	63	**111**	3	3	15	90
MS_11^(2)^	**21**	1	3	16	1	**37**	2	1	21	13	**29**	0	0	28	1	**14**	0	0	11	3
MS_12	**31**	1	1	28	1	**95**	1	1	30	63	**39**	1	0	15	23	**35**	0	0	17	18
**sum all**	**320 (135)**	47	49	177	47	**1430 (816)**	38	83	222	1087	**641 (392)**	24	32	177	408	**251**	5	13	90	143

^(1)^ This pair opened the contact window in night 3. Their data for night 3 were thus not included into the analysis;

^(2)^ These pairs vocalized at the male bedding; MS_07 = 1 song; MS_10 = 5 songs; MS_11 = 8 songs.

### Audio recording

Four ultrasound-microphones (condenser ultrasound-microphone CM16/CMPA, Avisoft Bioacoustics, Germany) were attached above the arena (two above each compartment), 50 cm above ground, 25 cm away from the separation wall, and evenly spaced above each compartment (Fig 1a and 1d). All four microphones were connected to a multichannel recording device (Avisoft UltraSoundGate 416, 4-channel). Recordings were made with a sampling rate of 500 kHz, with a depth of 16 bit using the Avisoft USGH Recorder software (Avisoft, Germany). For recordings, the “whistle tracking” option was enabled to automatically detect mouse USV. Recording was triggered by sounds within the range of 20–250 kHz, and a minimum duration of at least 10 ms. In order to record the whole USV sequence, a pre-trigger of 200 ms was applied, and each recording event lasted until 1 second after the last automatically detected whistle.

Some USVs were recorded simultaneously by the two microphones closest to the contact window (Fig 1a). In those cases we used the longer of two simultaneously recorded templates for further analysis and removed the other. The double recordings hindered the decision about which animal emitted a USV when both recording partners were in the contact region of their compartment but not about where the USV was recorded (*i*.*e*. in the contact region). We thus decided to treat each pair of females as one sample for statistical analysis rather than each individual female.

### Video recording

For video recordings a digital camera (Eneo, Video E. Hartig GmbH, Germany) was attached to the ceiling above the arena. Videos were recorded using the fg3xcap32 software of the TSE VideoMot2 software package (Version 7.01, TSE Systems International) with a frame-rate of 25 fps, and stored as avi-files on an external hard-disk. To not disturb the normal dark-light cycle of the animals, 4 red light-bulbs were mounted above the arena and switched on for video recordings during night time.

### Sound analysis

The time-frequency course of USVs was extracted from our recordings semi-automatically using the software Selena (Department of Animals Physiology, University of Tübingen, Germany). USVs were displayed as colour spectrograms using 256 Fast Fourier Transformation (FFT, Hann window) with an FFT overlap of 85% and 0.49 kHz zero-padding. For data extraction the program selects the pixel with the highest amplitude in each instantaneous FFT in a manually marked section of the spectrogram. The pre-selected pixels are then visually checked and manually corrected if necessary. Afterwards time, frequency and amplitude value of each pixel are exported to a csv-file.

The raw data were then processed with a custom-written Matlab routine as used in our last study [17]. We followed the definition of songs and syllables from Holy & Guo [16], but adjusted the temporal intervals separating songs or syllables to those suggested by visual inspection of our recordings (500 ms for songs and 10 ms for syllables, respectively). For each song and syllable we calculated several parameters (number of songs, song and syllable duration, number of syllables per song, syllable rate, frequencies, slope, and number of jumps and turning points; see also [17]). Based on those calculations we then assigned each syllable to one of 13 syllable types as described in our last study [17]. In short, high-frequency syllables were discriminated based on the number and orientation of frequency modulations, *i*.*e*. jumps and turns. This differentiation resulted in Simple syllables, *i*.*e*. syllables without any sudden frequency jumps or turning points, with three distinct syllable types; Turn syllables, *i*.*e*. syllables with one or more turning points (a turning point is a change in slope), with three distinct syllable types; and Jump syllables, *i*.*e*. syllables with one or more sudden frequency jumps with 7 distinct syllable types (see S2 Fig and [17]).

**Fig 2:**
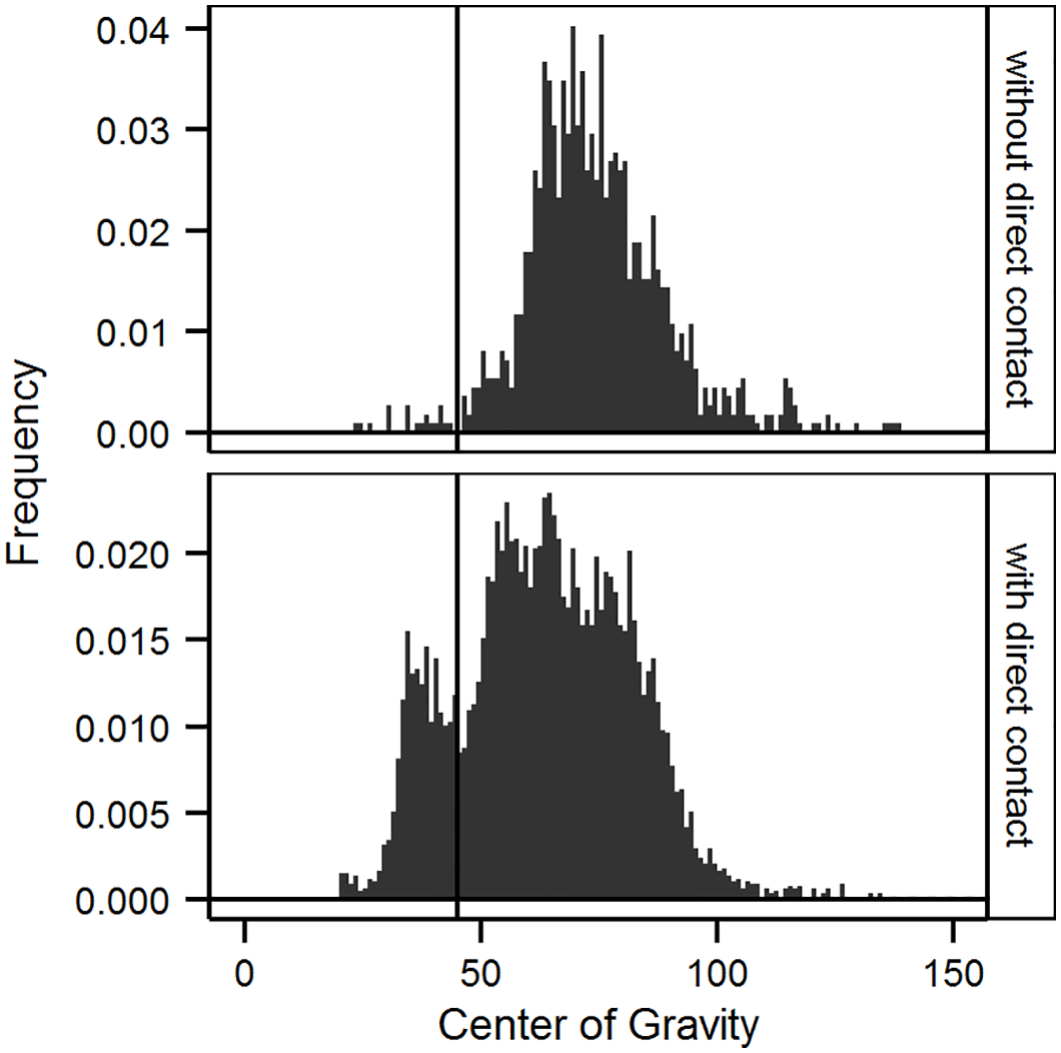
Frequency-distribution of the Center of Gravity of syllable frequency. Data for night 1 (without direct contact) are shown in the upper panel, data for nights 2–4 (with direct contact) in the lower panel. The vertical line is drawn at 45 kHz and separates low-frequency and high-frequency syllables.

We found a dichotomous distribution of the mean syllable frequency with a split at 45 kHz (Fig 2). Several of the syllables with a frequency below 45 kHz differed from the typical spectral shape of mouse USV, so that we decided to assign them to an additional syllable type (low-frequency syllables).

### Video scoring

Videos were scored manually, by inspecting those time frames where USVs were recorded and noting the current position of the singing mouse within the arena (Fig 1d). Video and audio recordings were synchronized manually by clapping two metal plates together while recording audio and video signal simultaneously before the onset of each experiment. We scored songs and presence of mice in five areas: the food region (FR), the nest region (NR), the contact region distant from the contact window (CR), close to the contact window (CW), and at the corners connecting both compartments (contact corners, CC), the latter representing indirect interaction possibilities via chemical cues (urine marks). The region around the male bedding was scored as region 6–male bedding (MB). For an overview of the different regions in the arena see Fig 1d.

To determine whether USVs were emitted more often during direct interaction at the contact window compared to more distant interaction, it was further distinguished between three possible type of encounters: (1) one individual at the contact window (solitary situations), (2) one individual at the contact window the other in the contact region (non-face to face encounters) or, (3) both individuals directly interacting at the contact window during singing (face to face encounters).

### Statistical analysis

Statistical analysis was separately done for (1) the number of songs uttered in each recording night and experimental region (SongCountData), (2) temporal song parameters, i.e. song duration and the number of syllables per song (SongParamData), (3) rate of syllables per second (RateData = SongParamData without songs containing only one syllable), and (4) SylData, which contained all measured syllable parameters as syllable duration, different frequency values, and the number of frequency modulations (jumps and turns).

Linear mixed models (function *LMER* of the R package lme4 [29]) were used to estimate the effect of recording night and context region or type of encounter at the contact window on the number of songs emitted. The maximal model included number of emitted songs as response variable and context night, context region or type of encounter, and their interaction as explanatory variables. Individual pairs of females were included as random factors. In order to find the minimal adequate model we used ANOVA statistics, and sequentially removed non-significant parameters.

For the statistical analysis of the qualitative data sets (SongParamData, RateData, and SylData) we used a PERMANOVA (PERmutational Multivariate ANalyses Of Variance; *Adonis* function of the R package *vegan* [30]). In order to correct for the repeated measurements design of this study, permutations were restricted to the level of the individual female dyads. Each data set (SongParamData, RateData, SylData) was analysed separately, with 5,000 permutations each.

For visualization of SylData, linear discriminant function analyses (function *LDA* of the R package *MASS* [31]) were run with either recording night or context region as grouping factor, and the first two linear discriminants were plotted. The linear discriminant analysis additionally provided a measure for the influence of each syllable parameter on the separation of recording nights, context regions, and types of encounters at the contact window.

For post-hoc comparisons we used non-parametric tests (Wilcoxon rank-sum and Wilcoxon signed rank test), because some of the parameters were not normally distributed (Shapiro-Wilks p < 0.05). Whenever necessary, significance levels were corrected for multiple testing via Bonferroni-correction.

In order to test whether syllable type usage differed between recording nights or context regions, X^2^-tests were applied. Because X^2^-tests cannot handle rare observations, syllables containing two frequency jumps were combined into the Two-Jump-Syllables (JMP) category for statistics. All statistical tests were carried out using R 3.1.2 [32].

## Results

In this study we focused on context specific vocalization of female mice. Our design allowed us to compare different contexts on a spatial scale (different context regions and types of contact) and a temporal scale (consecutive nights with different types of social interaction: none, with an unfamiliar female, with a familiar female, with or without a chemical male stimulus). We analysed the number of songs as well as temporal and spectral features of USVs in order to find out whether certain aspects of mouse USV changed with time and context.

**Fig 3:**
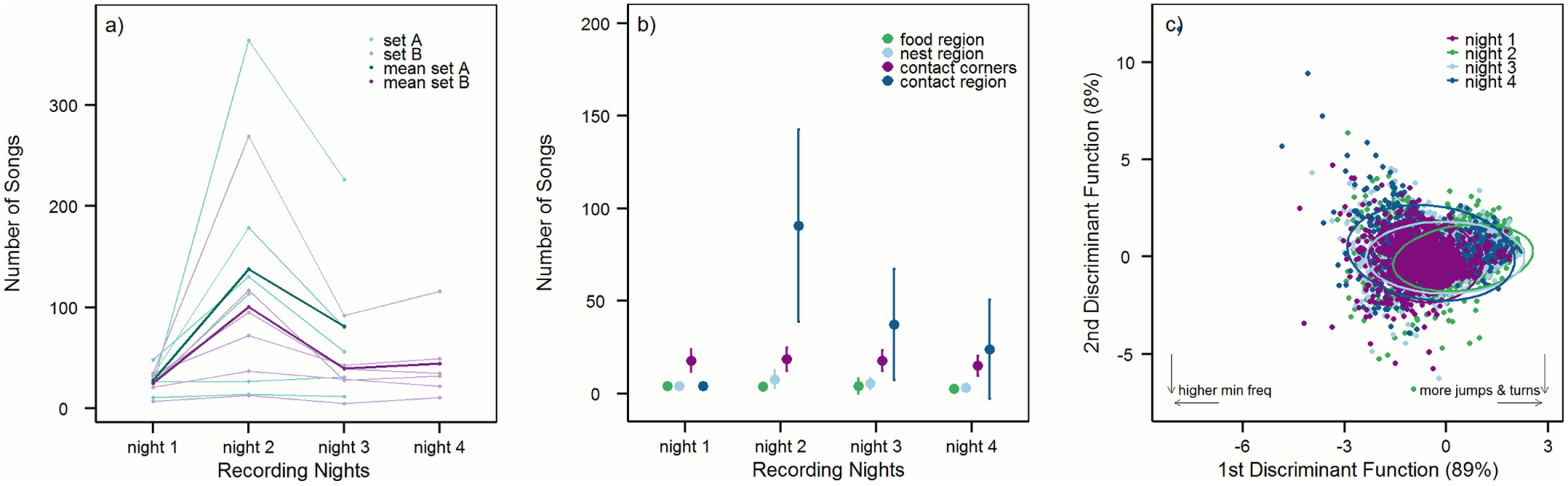
Songs and syllables in the different recording nights. a) The number of songs per night for each individual pair of females from sets A and B, as well as the mean number of songs per night in sets A and B. b) The mean number of songs in the different context regions in the four recording nights. c) The first two linear discriminant functions (LD) of syllable parameters in different recording nights. Ellipses show the 95% confidence interval around the group means. The contribution of the two linear discriminants is given below the axis. Arrows indicate the direction of the most influencing parameters on the separation of the data; for the loadings of these and the other syllable parameters see Table 3a.

In total 2656 songs, containing 14 different syllable types from 12 recording pairs were included in the analysis of song and syllable parameters. In general, we found strong differences in the number of USVs emitted by individual recording pairs in the different nights (Table 1). Especially in night 2, when the previously unfamiliar females met for the first time, song rates were much higher and differed significantly from song rates of nights 1 and 3 (Fig 3a, Tables 1 and 2a). In some pairs we recorded 100 times more songs in night 2 as opposed to night 1 (*e*.*g*. MS_05, MS_10). In others the number of USVs differed only marginally between nights 1 and 2 (*e*.*g*. MS_03, MS_04).

**Table 2:** Song parameters in different recording nights and context regions in night 1 and nights 2, 3, 4. Shown is the mean ± sd and the p-value of pairwise comparisons (Wilcoxon signed rank and rank sum tests) for the different recording nights (a), and context regions in night 1 (b) and nights 2, 3, and 4 (c).

		Mean of groups ± sd				Pairwise comparisons^(1)^					
	**Parameter**	**night 1**	**night 2**	**night 3**	**night 4**	**n1-n2**	**n1-n3**	**n1-n4**	**n2-n3**	**n2-n4**	**n3-n4**
**a)**	# of songs	26.67 ± 10.94	119.20 ± 107.20	58.27 ± 61.52	44.17 ± 37.43	***< 0*.*001***	***0*.*007***	0.171	***0*.*007***	0.036	0.528
	soDur	320.79 ± 381.97	263.65 ± 365.91	307.90 ± 409.49	301.97 ± 372.70	***< 0*.*001***	0.064	0.359	***0*.*003***	***0*.*003***	0.149
	# of syls^(2)^	3.50 ± 3.06	2.77 ± 2.57	3.06 ± 2.83	3.16 ± 2.68	***< 0*.*001***	0.031	0.402	0.037	***0*.*006***	0.224
		**FR**	**NR**	**CC**	**CR**	**FR-NR**	**FR-CC**	**FR-CR**	**NR-CC**	**NR-CR**	**CC-CR**
**b)**	# of songs	3.92 ± 2.94	4.08 ± 2.88	17.7 ± 9.66	3.92 ± 2.61	0.893	0.019	1.000	0.023	0.851	0.031
	soDur	270.27 ± 423.52	126.12 ± 251.53	419.41 ± 393.50	202.89 ± 281.76	0.021	***< 0*.*001***	0.378	***< 0*.*001***	0.195	***< 0*.*001***
	# of syls	2.92 ± 3.03	1.94 ± 2.14	4.29 ± 3.18	2.70 ± 2.53	0.013	***< 0*.*001***	0.453	***< 0*.*001***	0.138	***< 0*.*001***
**c)**	# of songs	6.70 ± 5.77	10.67 ± 11.11	40.75 ± 24.06	136.5 ± 148.2	0.036	***0*.*003***	***< 0*.*001***	***0*.*007***	***0*.*003***	0.009
	soDur	144.25 ± 142.29	129.45 ± 208.96	500.88 ± 493.89	229.90 ± 326.63	0.089	0.362	***< 0*.*001***	***< 0*.*001***	***< 0*.*001***	***< 0*.*001***
	# of syls	1.99 ± 1.08	1.70 ± 1.20	4.70 ± 3.60	2.47 ± 2.17	0.009	0.585	***< 0*.*001***	***< 0*.*001***	***< 0*.*001***	***< 0*.*001***

^(1)^ Significance level after Bonferroni correction: p < 0.0083;

^(2)^ syls = syllables.

The number of songs emitted in the repetition experiments with the same individual dyads of mice after some time correlate significantly with those in the first experimental trials (Pearson’s correlation coefficient = 0.97, p = 0.006; Table 1 and S1 Fig).

### Vocalization in different recording nights

Analysing SongCountData data by means of linear mixed models, we identified a strong influence of context region and recording night on the quantity of vocalizations (S2 Table).

With pairwise comparisons we found that the highest number of vocalizations was linked to the contact region in night 2 (Fig 3a and 3b, Table 2a), when the two previously unfamiliar recording partners met for the first time, and that the number of songs decreased in nights 3 and 4, when recording partners were more familiar (Fig 3a and Table 2a). The linear model fitted better, when night 3 and 4 were combined as opposed to the model with nights 3 and 4 separated (S3 Table), showing that the male bedding added in either night 3 (set A) or night 4 (set B) had no influence on SongCountData.

Recording night also had a significant influence on song and syllable parameters, but not on syllable rate (PERMANOVA comparing between recording nights: SongParamData: F(3) = 5.32, p = 0.003; SylData: F(3) = 181.75, p = 0.0002; RateData: F(3) = 1.39, p = 0.249).

Pairwise comparisons showed that songs recorded in nights with direct contact were on average shorter than songs from night 1, although this difference was only significant in the comparison of night 1 and night 2 (Table 2a). Further, songs from nights with direct contact contained significantly less syllables than songs from night 1 (Table 2a). Syllables from nights 2 and 3, but not night 4, were shorter than syllables from night 1, had a lower mean frequency and were less complex, *i*.*e*. contained less jumps and turning points (Fig 3c and Table 3a). Also between nights 2 and 3 and nights 2 and 4, we found differences in certain song and syllable features, but nearly none between nights 3 and 4 (Tables 2a and 3a).

**Table 3:** Syllable parameters in different recording nights and context regions in night 1 and nights 2, 3, 4. Shown is the mean ± sd for each night or region, the loadings of the first 3 linear discriminant functions, and the p-value of pairwise comparisons (Wilcoxon rank sum tests) for the different recording nights (a), and context regions in night 1 (b) and nights 2, 3, and 4 (c).

		Mean of groups ± sd				Loadings			Pairwise comparisons^(1)^					
	**Parameter**	**night 1**	**night 2**	**night 3**	**night 4**	**LD1**	**LD2**	**LD3**	**n1-n2**	**n1-n3**	**n1-n4**	**n2-n3**	**n2-n4**	**n3-n4**
**a)**	sylDur	40.71 ± 33.67	22.24 ± 23.17	29.79 ± 28.70	41.13 ± 37.85	-0.019	0.041	-0.018	***< 0*.*001***	***< 0*.*001***	0.277	***< 0*.*001***	***< 0*.*001***	**0.006**
	staFreq	78.25 ± 16.84	60.65 ± 19.44	68.28 ± 19.90	72.49 ± 21.18	-0.008	0.017	0.028	***< 0*.*001***	***< 0*.*001***	***< 0*.*001***	***< 0*.*001***	***< 0*.*001***	0.478
	slope	0.01 ± 0.78	-0.03 ± 0.93	0.00 ± 0.86	0.03 ± 0.78	-0.071	-0.093	0.033	***0*.*001***	0.980	0.812	***0*.*001***	0.009	0.203
	minFreq	65.08 ± 15.40	53.11 ± 16.47	59.22 ± 17.41	60.16 ± 17.41	-0.047	0.229	0.276	***< 0*.*001***	***< 0*.*001***	***< 0*.*001***	***< 0*.*001***	***< 0*.*001***	0.802
	freqBand	24.95 ± 17.13	15.54 ± 14.24	18.71 ± 16.07	24.34 ± 18.27	-0.040	0.020	0.048	***< 0*.*001***	***< 0*.*001***	0.628	***< 0*.*001***	***< 0*.*001***	0.100
	freqCOG	74.31 ± 14.91	60.44 ± 17.39	67.36 ± 17.89	69.90 ± 18.11	0.041	0.046	-0.093	***< 0*.*001***	***< 0*.*001***	***< 0*.*001***	***< 0*.*001***	***< 0*.*001***	0.849
	jumps	0.77 ± 1.01	0.32 ± 0.73	0.44 ± 0.81	0.60 ± 0.87	0.090	-1.342	-0.471	***< 0*.*001***	***< 0*.*001***	***< 0*.*001***	***< 0*.*001***	***< 0*.*001***	0.330
	turns	2.04 ± 2.28	1.08 ± 1.62	1.31 ± 1.80	1.78 ± 1.99	0.076	-0.365	0.512	***< 0*.*001***	***< 0*.*001***	0.044	***< 0*.*001***	***< 0*.*001***	0.117
		Proportion of trace:				0.894	0.084	0.022						
		**FR**	**NR**	**CC**	**CR**	**LD1**	**LD2**	**LD3**	**FR-NR**	**FR-CC**	**FR-CR**	**NR-CC**	**NR-CR**	**CC-CR**
**b)**	sylDur	39.46 ± 31.80	26.16 ± 20.37	43.70 ± 35.13	35.96 ± 31.75	-0.019	-0.032	-0.009	***< 0*.*001***	0.095	0.190	***< 0*.*001***	0.053	***< 0*.*001***
	staFreq	77.50 ± 14.90	76.33 ± 22.38	78.91 ± 15.78	76.82 ± 19.50	0.013	0.012	0.003	0.446	0.922	0.240	0.312	0.946	0.121
	slope	-0.11 ± 0.77	-0.08 ± 0.80	0.04 ± 0.76	0.01 ± 0.85	-0.349	0.401	0.993	0.467	0.299	0.141	0.059	0.082	0.487
	minFreq	65.26 ± 15.04	66.38 ± 20.95	64.77 ± 13.91	65.71 ± 18.63	-0.114	0.046	-0.050	0.723	0.516	0.730	0.566	0.550	0.833
	freqBand	21.53 ± 15.86	17.51 ± 13.07	27.23 ± 17.46	21.26 ± 16.52	-0.089	0.063	-0.003	0.087	***< 0*.*001***	0.930	***< 0*.*001***	0.170	***< 0*.*001***
	freqCOG	74.18 ± 14.78	74.08 ± 20.60	74.33 ± 13.45	74.53 ± 17.69	0.087	-0.068	0.041	0.994	0.772	0.698	0.925	0.810	0.731
	jumps	0.73 ± 1.11	0.33 ± 0.55	0.86 ± 1.03	0.65 ± 1.01	-0.075	-1.608	0.348	0.013	0.022	0.716	***< 0*.*001***	0.025	***0*.*004***
	turns	1.90 ± 2.26	1.54 ± 1.74	2.20 ± 2.35	1.68 ± 2.20	0.282	0.745	-0.311	0.436	0.065	0.361	***0*.*007***	0.952	***0*.*002***
		Proportion of trace:				0.760	0.221	0.019						
**c)**	sylDur	23.77 ± 18.16	17.82 ± 13.77	43.45 ± 34.66	17.55 ± 17.61	-0.022	0.012	-0.018	***0*.*001***	***< 0*.*001***	***< 0*.*001***	***< 0*.*001***	0.158	***< 0*.*001***
	staFreq	74.48 ± 21.01	61.36 ± 19.33	77.34 ± 17.06	56.44 ± 17.64	-0.025	-0.008	0.105	***< 0*.*001***	0.190	***< 0*.*001***	***< 0*.*001***	***< 0*.*001***	***< 0*.*001***
	slope	0.21 ± 0.91	-0.04 ± 1.04	0.04 ± 0.77	-0.05 ± 0.94	-0.172	-0.206	1.062	0.099	***0*.*006***	***< 0*.*001***	0.429	0.032	***< 0*.*001***
	minFreq	66.25 ± 18.17	56.44 ± 18.85	63.83 ± 14.76	50.69 ± 16.31	-0.063	0.029	-0.231	***< 0*.*001***	0.016	***< 0*.*001***	***< 0*.*001***	***< 0*.*001***	***< 0*.*001***
	freqBand	18.39 ± 15.90	12.51 ± 12.30	25.8 ± 17.60	12.85 ± 11.90	-0.037	0.021	-0.054	***0*.*001***	***< 0*.*001***	***< 0*.*001***	***< 0*.*001***	0.617	***< 0*.*001***
	freqCOG	74.86 ± 18.71	62.7 ± 19.48	73.71 ± 14.59	57.43 ± 16.85	0.049	-0.062	0.119	***< 0*.*001***	0.144	***< 0*.*001***	***< 0*.*001***	***< 0*.*001***	***< 0*.*001***
	jumps	0.35 ± 0.60	0.16 ± 0.51	0.77 ± 1.00	0.18 ± 0.53	-0.127	-0.256	-0.682	***< 0*.*001***	***< 0*.*001***	***< 0*.*001***	***< 0*.*001***	0.404	***< 0*.*001***
	turns	0.85 ± 1.23	0.62 ± 0.97	2.02 ± 2.22	0.81 ± 1.23	0.114	0.320	0.314	0.165	***< 0*.*001***	0.988	***< 0*.*001***	0.024	***< 0*.*001***
		Proportion of trace:				0.977	0.020	0.003						

^(1)^ Significance level after Bonferroni correction: p < 0.0083

Additionally, we found a difference in the distribution of the mean syllable frequency when comparing nights with and without potential direct contact. The mean syllable frequency in night 1 was normally distributed, with a mean at 74.31 ± 14.91 kHz, whereas the distribution of the mean syllable frequency was dichotomous in nights 2–4, separating the syllables into one group with a mean frequency above, and one with a mean frequency below 45 kHz (Fig 2).

### Vocalization in different context regions

**Fig 4:**
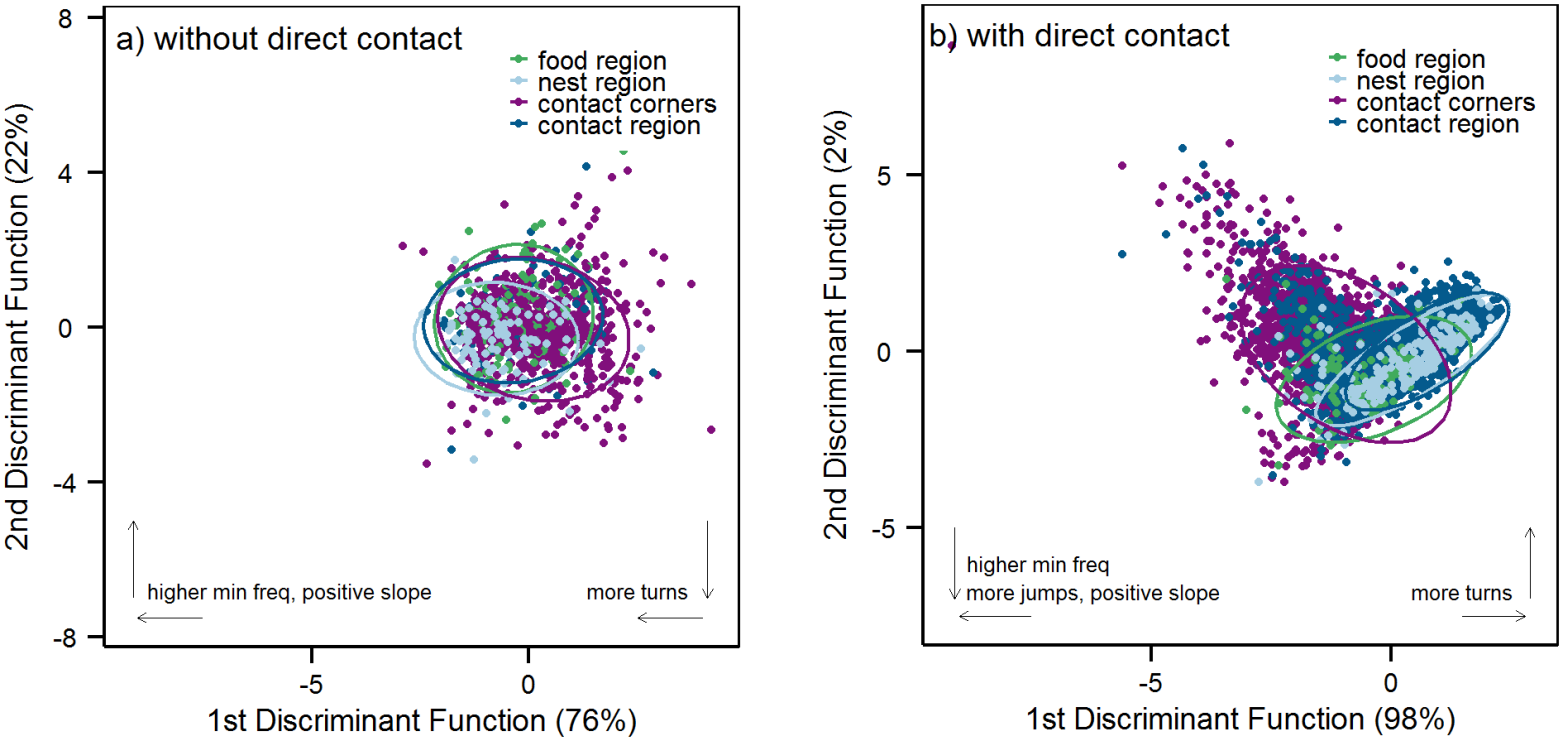
Syllables in different context regions. a) The first two linear discriminant functions (LD) of syllable parameters in different context regions in nights without direct contact (night 1). b) The first two linear discriminant functions (LD) of syllable parameters in different context regions in nights with direct contact (nights 2, 3, 4). Ellipses in the scatterplots show the 95% confidence interval around the group means. The contribution of the two linear discriminants is given below the axis. Arrows indicate the direction of the most influencing parameters on the separation of the data; for the loadings of these and the other syllable parameters see Table 3b and 3c.

We further compared song and syllable features between context regions, separately for night 1 and nights 2–4. We found a strong influence of the different context regions on song and syllable parameters in night 1 (PERMANOVA-SongParamData: F(3) = 7.13, p = 0.0002; PERMANOVA-RateData: F(3) = 9.18, p = 0.0002; PERMANOVA-SylData: F(3) = 5.31, p = 0.0002) and in nights 2–4 (PERMANOVA-SongParamData: F(3) = 100.04, p = 0.0002; PERMANOVA-SylData: F(3) = 441.95, p = 0.0002). Differences between context regions were mainly linked to the two social regions (the contact region and the contact corners, Fig 4a and 4b).

In night 1, the night without potential direct contact, the number of vocalizations was highest in the contact corners (see Fig 3b and Tables 1 and 2b), and also in terms of song and syllable parameters USVs in the corners differed from USVs elicited elsewhere (Fig 4a and Tables 2b and 3b). Contact corner songs in night 1 were longer than songs recorded in food, nest, or contact region and contained a higher number of syllables per song (Table 2b). Syllables from the contact corners were also longer than syllables from the other regions (Table 3b), and had a higher number of jumps and turning points, and covered a significantly broader frequency band. Songs and syllables from the food, nest, and contact region differed only in a few parameters (Fig 4a and Tables 2b and 3b).

In nights 2–4 (direct contact possible), vocalization rates were highest in the contact region (Fig 3b, Tables 1 and 2c). Nevertheless, vocalization in the corners was still used more often than vocalization in nest and food context (Table 1 and Fig 3b). Linear discriminant analysis and post-hoc tests showed that song and syllable parameters were highly specific to the two types of social interaction: direct (in the contact region) and indirect (in the contact corners) (Fig 4b and Table 3c). The most striking characteristic of USVs from the contact region was their low frequency, due to a high amount of the low-frequency syllables (S2 Fig) we recorded from the second night on (Fig 2). Further, syllables from the contact region in general were short, had a negative slope, and contained only few frequency jumps, and thus had a narrow frequency bandwidth (for all values of syllable parameters see Table 3c). Songs from the contact region were short (for all values of song parameters see Table 2c) and contained relatively few syllables per song. In contrast to this, songs recorded in the contact corners were long and contained a high number of syllables per song. Syllables from the contact corners were long, had high frequency values, a positive slope, and contained a high number of frequency jumps and turning points. All pairwise comparisons of contact corners and contact region songs and syllables were highly significant (p < 0.001, Tables 2c and 3c), showing that USVs emitted in the two social regions differed strongly in all measured parameters (Fig 4b). Those differences can be explained by the usage of certain syllable types in the contact region and the contact corners. More jump and simple-up syllables were used in the contact corners, and more low-frequency, and simple-down syllables in the contact region (X^2^ (12) = 2277.8, p < 0.001; see S2 Fig).

We also found differences in food and nest region USVs in nights 2–4. Vocalizations from the nest region shared some features with contact region syllables, but had significantly higher frequency values and had a more positive frequency slope (Fig 4b and Table 3c). Food region syllables had frequency values comparable to syllables from the contact corners, whereas other syllable parameters were significantly different in food region and contact corners (Table 3c).

Only very few songs were recorded at the male bedding (overall 14 songs from 3 pairs). Those were comparable to songs from the contact corners in most parameters (S7 Table), but the low number of vocalizations impeded any statistical comparison.

### Encounters at the contact window

**Fig 5:**
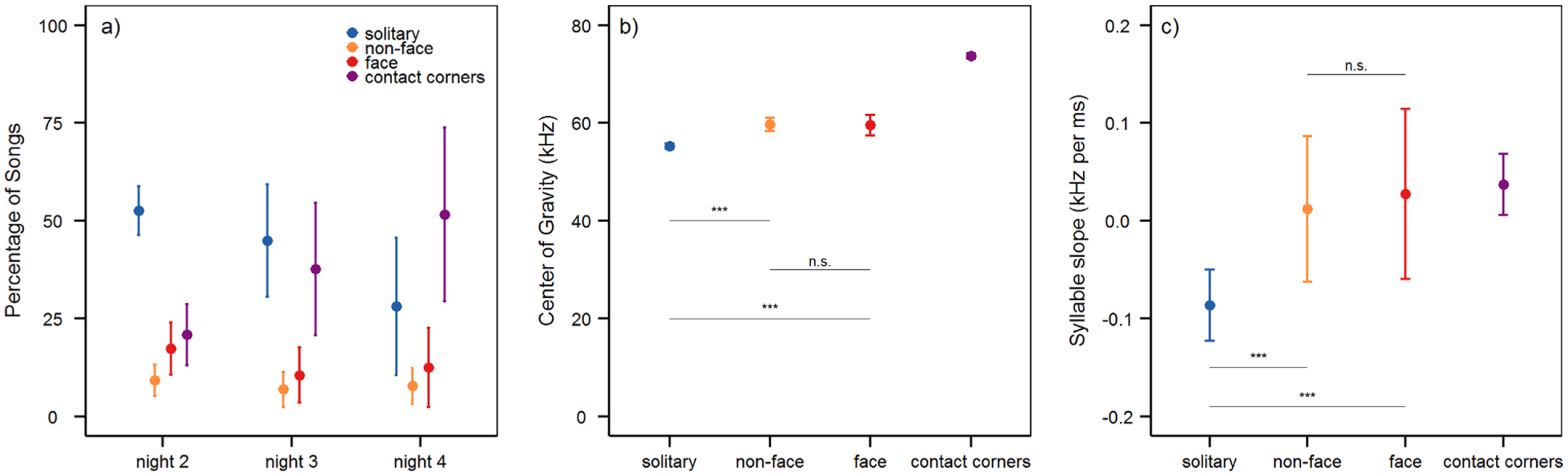
Songs and syllables in different encounters at the contact window. a) The amount of songs recorded during different encounters at the contact window (solitary situations, face to face and non-face to face encounters), and in the contact corners in percent, shown separately for nights 2, 3, and 4. Given is the mean ± the 95% confidence interval for each group. b) The mean (± 95% confidence interval) of the Center of Gravity in syllables from solitary situations, face to face and non-face to face encounters in the contact region, and contact corner syllables. c) The mean (± 95% confidence interval) of the frequency slope in syllables from solitary situations, face to face and non-face to face encounters, and contact corner syllables. Abbreviations for the different types of encounters are: solitary situations = solitary; face to face encounters = face; non-face to face encounters = non-face. The test statistics for pairwise comparisons can be found in Table 4.

We further compared vocalization in the contact region between different type of encounters: face to face encounters (both individuals interacting at the contact window), non-face to face encounters (one individual at the window, the other somewhere in the contact region), and solitary (only one individual at the contact window). The number of vocalizations differed according to the type of encounter and according to recording night (S5 Table), and was significantly higher in the solitary situations as opposed to non-face to face or face to face encounters (Fig 5a and Table 4). Also the syllables in the solitary situation had significantly lower frequency values (Fig 5b), were significantly shorter, and had a more negative slope than syllables from non-face to face or face to face encounters (Fig 5c). The number of jumps and turns was higher for vocalizations emitted in non-face to face encounters, and lower for face to face encounters compared to solitary situations, this difference was however not significant. Neither number of vocalizations nor any song or syllable parameters differed between non-face to face and face to face encounters, except the number of turning points, which was higher for vocalizations from non-face to face as opposed to face to face encounters (Table 4). See Table 4 for the values of all parameters and the test results of all pairwise comparisons.

**Table 4:** Song and syllable parameters in different encounters at the contact window. Shown is the mean ± sd for each type of encounter, the loadings of the first 2 linear discriminant functions, and the p-value of pairwise comparisons (Wilcoxon signed rank and rank sum tests). Abbreviations for pairwise comparisons are: sol = solitary, nf = non-face to face, ftf = face to face.

	Mean of groups ± sd			Loadings		Pairwise comparisons^(1)^		
Parameter	solitary	non-face to face	face to face	LD1	LD2	sol-nf	sol-ftf	nf-ftf
# of songs	37.69 ± 45.20	12.43 ± 15.40	15.74 ± 19.65			***< 0*.*001***	***< 0*.*001***	0.084
soDur	227.46 ± 312.77	240.64 ± 338.10	165.53 ± 266.72			0.669	0.008	0.020
# of syls	2.46 ± 2.08	2.49 ± 2.22	2.01 ± 1.63			0.982	0.002	0.020
sylDur	15.89 ± 15.11	17.77 ± 16.93	17.30 ± 11.70	0.035	0.043	***< 0*.*001***	***< 0*.*001***	0.244
staFreq	54.27 ± 16.32	58.57 ± 18.59	57.78 ± 20.86	-0.012	-0.108	***< 0*.*001***	***0*.*007***	0.427
slope	-0.09 ± 0.96	0.01 ± 0.97	0.03 ± 0.86	0.204	-0.595	0.023	***< 0*.*001***	0.140
minFreq	48.71 ± 15.04	53.02 ± 17.59	53.53 ± 20.13	0.059	0.116	***< 0*.*001***	***< 0*.*001***	0.893
freqBand	12.13 ± 11.36	12.78 ± 11.34	11.32 ± 9.82	-0.009	0.032	0.076	0.688	0.116
freqCOG	55.31 ± 15.57	59.76 ± 17.64	59.57 ± 20.70	0.000	-0.032	***< 0*.*001***	***< 0*.*001***	0.748
jumps	0.14 ± 0.46	0.18 ± 0.48	0.12 ± 0.39	0.333	-0.232	0.030	0.608	0.066
turns	0.76 ± 1.13	0.82 ± 1.15	0.61 ± 0.95	-0.355	-0.810	0.401	0.021	***0*.*013***
	Proportion of trace:			0.819	0.181			

^(1)^ Significance level after Bonferroni correction: p < 0.016.

## Discussion

In the present study we focused on context related vocalization in female mice, where individual females were confronted with a female conspecific. Conspecifics could have been noticed either indirectly (using chemical cues) since the beginning of each experiment, or directly (using visual, chemical, auditory, and tactile cues), possible from the second night on, when the contact window was opened. In this study female mouse vocalization was mainly triggered by social stimuli. Clear differences could be observed in song and syllable characteristics between vocalizations that were recorded in indirect and those recorded in direct interactions (contact corners vs. contact region, and face to face or non-face to face encounters vs. solitary situations).

### Vocalization is mainly triggered by social interactions

Our data show that female mice vocalize mainly, but not exclusively, in social contexts. During the first night most USVs were emitted in the contact corners (Fig 3b). Video recordings suggested that these corners were frequently used by all females while urinating, which could be confirmed after each experiment. As we can exclude mate attraction as source for urine marking behaviour, territoriality may have evoked scent marking. Scent is a strong communication signal in mice (*e*.*g*. in the context of mate choice and MHC [33, 34], territoriality (*e*.*g*. [13], reviewed in [35]), social status (*e*.*g*. [36, 37])) so it is very likely that females noticed their recording partners via scent already without the possibility to hear or see them. After opening the contact window in the second night vocalization levels were higher as opposed to night 1. The majority of USVs was emitted in the contact region where direct interaction with the recording partner was possible, followed by vocalization in the contact corners. Direct interaction in the contact region thus represented a more motivating stimulus for vocalizations than did indirect perception of the other female in the contact corners. Also in the non-social contexts some vocalization were emitted, but the amount was far lower than in the social contexts. A social effect on vocalization has already been shown for male and female mice of the genus *Peromyscus* in the wild [23, 38]. Kalcounis-Rueppell and colleagues could show that mice vocalized the most, when interacting with a conspecific and this effect was strongest for the communication between neighbouring females. Also in male lab mice the social effect on vocalization has already been shown (*e*.*g*. [26]). The possibility to communicate with a social partner while another social stimulus was provided (male bedding) did not result in a higher level of vocalization (Fig 3a).

Although most dyads vocalized more in night 2 as opposed to night 1, we found strong inter-pair differences in the number of USVs recorded (Fig 3 and S1 Fig). This observation fits other studies showing that the propensity to sing seems to be a personal characteristic of an individual mouse [17].

### Female USV is affected by familiarization

In our experiments we simulated encounters of unfamiliar female mice as they might occur between females from neighbouring nests or territories in the wild [23]. As described in the Material and Methods section, recording partners in our experiments were not complete strangers because they were housed in close proximity to each other. Nevertheless, they were unfamiliar in the sense that they never directly interacted with each other and thus were not able to establish any kind of social relationship before the experiments. Our data clearly show that social familiarity reduced the number of USVs emitted by a pair of females (Fig 3a). The contact with an unfamiliar female in night 2 elicited more USVs than did the prolonged contact with the same female in nights 3 and 4, respectively. This finding is in line with previous studies on female USV [22, 25]. Intriguingly, we found that the number of USVs emitted by the pairs that were exposed to two trials was highly correlated between the trials. Further, we also observed the same familiarity effect as in the first trials (see S1 Fig). Re-establishing interactions between familiar mice thus elicited as many USVs as did the first contact with an unfamiliar individual. This is in line with findings from Moles and D’Amato [24]. They found that the amount of female USV in re-encounters after 24 hours of separation was comparable to when they met for the first time. It has been shown that female mice exhibit non-random social preferences for other females and that this choice has the potential for direct fitness consequences [10]. Potentially, the number of USVs recorded from two interacting females could express their level of social compliance, as already proposed by Moles *et al*. [25]. However, our data do not allow testing this hypothesis any further.

Song and syllable parameters were also affected by social familiarity, as we found differences between night 2 and nights 3 and 4 (Fig 3c). This is to our knowledge the first time that the effect of social familiarity was not only shown in the number of songs, but also in spectral and temporal USV parameters. The familiarity effect was mainly linked to songs recorded in the contact region, where the number of USVs was highest in night 2, when the previously unfamiliar females met for the first time, and lower in nights 3 and 4, when females were already familiar. Possibly, the number of USVs from the contact region decreased in nights 3 and 4 additionally because the animals spent less time in the contact region; as we did not quantify how much time the animals spent in each arena region in the different recording nights, we cannot differentiate between those two effects. The conclusion, however, is the same: The animals vocalized the most in the contact region during the second night. The number of contact corner vocalizations was relatively stable over all recording nights, showing that they were not directly influenced by the possibility for direct interaction or by social familiarity.

Von Merten *et al*. did not find a familiarity effect when comparing the number and spectral and temporal parameters of USVs recorded in two successive recording nights [17]. They also recorded wild female mice in a comparable, but much smaller setup, but they did not test for differences between context regions or for differences between different social situations, so results might not be comparable.

### Temporal and spectral USV characteristics used in non-social vs. social situations

A study in male mice showed that vocalization under complete social isolation differed from vocalizations recorded under social conditions [26]. Our experiment confirmed these results in a more natural setup: Comparing wild female mice between a more isolated vs. a more social situation, we found differences in song and syllable characteristics depending on the context USVs were emitted in (Fig 4).

Comparisons of vocalization parameters between different non-social contexts (food and nest context) did not reveal context-specific parameters. USVs from food, nest, and “non-active” contact region differed only marginally in night 1, showing that USVs in the absence of conspecifics were not highly specific to single non-social stimuli. In nights with direct social contact we found significant differences also between vocalization in the nest and food region. This could mean that mice use USVs specific to those two contexts in the presence of another conspecific. In rats it was shown that they use specific USVs in the context of feeding during male-male interactions [28]. Nest and food region USVs in our experiment might however as well differ from each other because of their proximity to either the contact region or the contact corners (see Fig 1). Food region syllables from nights with direct social contact shared several characteristics with syllables from the contact corners, whereas nest region syllables shared characteristics with contact region syllables, indicating that vocalizations in nest and food regions might have been influenced by their proximity to the social regions in nights with direct social contact. The number of songs when male bedding was provided (14 songs of three pairs) was very low. Nevertheless, emitted USVs seem to have certain characteristics in common with USVs from the contact corners (*e*.*g*. a high frequency and a high number of frequency modulations, see S7 Table), and different from contact region USVs (for contact region parameters see Table 3c).

### USV characteristics from different social situations

While we found only little differences in USV between non-social situations, we found strong differences when comparing different social contexts: First, USVs from the contact corners vs. USVs from the contact region, and second, vocalizations from the contact window, which were further subdivided into face to face, non-face to face, and solitary vocalizations.

#### Directed vs. undirected communication

The contact region and contact corner songs from our study can be classified as directed and undirected vocalizations, as they are known from birdsong [39, 40] and from male mice [27]. Undirected vocalizations address any conspecific nearby, and are often sung alone, whereas directed vocalizations address a certain individual. Songs recorded in the contact region could resemble directed vocalizations, that are directed towards the recording partner, whereas vocalizations from the contact corners could address any conspecific in the surroundings, and do not depend on the physical presence of a conspecific. Vocalizations from the contact corners and the contact region differed in all measured song and syllable parameters (Fig 4c and Table 3c). The most notable effect in these comparisons was the occurrence of low-frequency syllables, which were linked to a possible direct interaction in the contact region. Furthermore, songs emitted in the contact region contained few syllables, and syllables with a more negative slope, and few frequency modulations, *i*.*e*. frequency jumps and turning points. In contrast, syllables emitted in the contact corners had high frequencies, a positive slope, and a high number of frequency jumps. Our results confirm recent findings in male lab mice which produce two different types of USVs depending on whether individuals encounter real conspecifics or female urine only, thus reflecting directed and undirected communication contexts [27]. Like in our study, those males also used songs with higher frequencies and more complex syllable types in undirected communication (presence of female urine), as opposed to directed communication (presence of real females). It seems counterintuitive that mice use more complex vocalizations with higher frequencies in undirected song and simpler vocalization with low frequencies in directed song. Communication over longer distances is more energy consumptive as, firstly, high frequencies do not reach far due to attenuation [41], and secondly, complex syllable types (e.g. with frequency jumps) are presumably more costly to produce than simple ones [27]. Using such a costly signal suggests that undirected song has an important function in mouse communication. Males use their complex, highly energetic songs only in the presence of female urine, and thus only in the mating context. They do not vocalize in the presence of male urine [27]. Under natural conditions, a male could emit a complex, highly energetic song when he encounters fresh female urine in order to call the female which is presumably still nearby. Females in the area could evaluate the complex song to gather information about the singer that helps them to decide whether to approach him or not [19, 27, 42]. Once a female is attracted by the complex male song and approaches, it might be sufficient to switch to the more simple song to keep her close while she can access and evaluate other cues, like scent, which might signal information about the male important for mate choice and/or immune status [33, 34]. The complex, energetic male songs could thus be far-distance signals, which are used before other more relevant cues like scent are recognizable. Female undirected songs could have a similar affiliative function as shown for male undirected songs [27] and could motivate conspecifics to approach and investigate the singer. On the other hand, female undirected songs could indicate the boundary to a neighbouring territory [23]. Similar to the male songs, other conspecifics in the area, be it males or females, could evaluate the complex female song in order to decide whether to approach, or not.

Females have been shown to also vocalize in male-female interactions [17, 23, 38, 43], and in a recent study, we found that also male mice choose their female partner (Linnenbrink & von Merten, unpublished data). Thus, undirected female song could possibly be a basis for male mate-choice, like male undirected song for female mate-choice [19, 27].

Undirected vocalization in our study was mostly observed when females deposited urine. These songs could thus engage other individuals to investigate the fresh urine marks to gather further information about the female, before getting into closer contact with her. As we found that females, unlike males [19] used their complex USVs in the presence of same-sex cues, the undirected female songs might also convey important information for other females, for example information about oestrus or reproductive state that could influence female social preferences [25] and might be interesting in terms of communal nesting and pup rearing [8, 9, 10, 11]. Further, in combination with urine marks, undirected songs might also be used as signals for territoriality [23].

#### Communication at the garden fence

We further disentangled the context of vocalizations from the observed directed songs, distinguishing between songs in three different types of encounters at the contact window: First, one mouse alone at the open window (solitary situations), second, one mouse at the open window, their recording partner somewhere in the contact region (non-face to face encounters) or, third, both mice at the open contact window (face to face encounters). Most of the songs from the contact region occurred in the second experimental night, when the previously unfamiliar females met for the first time (Fig 3b). In the following nights the number of songs in the contact region decreased. This is in line with the hypothesis of familiarization [22], as in the first encounters females might have established a dominance relationship [25, 44]. During face to face encounters, when both females met at the open contact window, we could observe both friendly behaviour (e.g. sniffing each other), as well as aggressive behaviour and high states of arousal (bites through the window and tail rattling [45, 46, 47]). Both male and female mice have been shown to establish stable social hierarchies [48, 49]. In our setup however, we were not able to tell if a hierarchy was established and, if so, which animal was the “winner” or “loser” of the interaction. Thus, we cannot decide if USVs emitted during face to face encounters are a signal for aggression, submission and/or general arousal.

The majority of directed songs were emitted in solitary situations, recorded when one animal was alone at the contact window (Fig 5a). This is contrary to other recent findings, where more vocalizations were recorded in situations of two interacting females [22, 23]. Solitary USVs, *i*.*e*. songs recorded when one animal was alone at the open contact window could be used to call the recording partner, announce what is termed “approaching in a friendly manner” [25], or be used as territorial calls to warn the other female not to enter the own territory [23, 38].

According to the Motivation-Structural rule hypothesis [50] a low vocalization frequency is linked to aggression and negative emotional states. This has been confirmed for rats, mice, and hamsters (*e*.*g*. [26, 51, 52, 53]). Accordingly, the low frequency of directed songs as we recorded them in the contact region could be a sign for aggression and negative emotions. Most studies on female USV however argue that social contact with another female is a rewarding stimulus rather than a negative situation [25]. Low-frequency vocalizations do not necessarily signal aggression or negative motivational states as studies in birds [54] and mice show [27]. Additionally, negative emotional states are predicted to elicit only few vocalizations [26]. We nevertheless recorded a high number of vocalizations in the contact region, a lot of them being of low frequency USV.

If the low vocalization frequency of directed songs from the contact region was a signal for aggression we would have expected that low frequency USV were mostly recorded in the condition with the highest potential for aggression, *i*.*e*. when both animals were interacting at the contact window. This was not the case (Fig 5b). In contrast, most low frequency USVs were emitted when one mouse was alone at the contact window. This fits to a function of low frequency USV in calling for a more distant conspecific, as low frequencies can travel further distances.

## Conclusions

We could show conclusively, that female house mice do vocalize and this preferably when they meet a conspecific. Our results suggest that female USV is linked to familiarization and potentially hierarchy establishment, and that the number of USVs recorded during female-female encounters might be a pair-related trait expressing social preferences. Furthermore, female vocalization differs if used as directed communication towards another female or undirected communication, in terms of marking a territory coupled with urination. This supports the notion that vocal communication in house mice is an important factor not only in males, but also in females, and that it is clearly context dependent. Further, mouse communication is driven by a reasonable and yet underestimated amount of complexity that also involves the interplay between different sensory signals, like chemical and auditory cues. Further studies will be necessary to study this complexity in more detail.

## Supporting Information

S1 Fig Results of the repetitions of five recording pairs. a) The number of songs recorded from each pair of females in the different recording nights, shown for the first and the second trial. The respective repetition for each pair is indicated by an “r” after the pair name. b) The total number of songs recorded from each pair of females in trial 1 and trial 2.
(TIFF)

S2 Fig Syllable types used in the contact corners and the contact region in nights 2–4. Shown is the proportion of each syllable type in percent. Syllable types are: SFL = Simple-Flat, SUP = Simple-Up, SDN = Simple-Down, TUD = Turn-Up-Down, TDU = Turn- Down-Up, TRS = Turn-Multi, JEU = Jump-Early-Up, JED = Jump-Early-Down, JLU = Jump-Late-Up, JLD = Jump-Late-Down, JMP = Two-Jump, JPS = Jump-Multi, LFS = Low-Frequency-Syllables. For a detailed description of each syllable type see [17].
(TIFF)

S1 File Song and syllable parameters of each recorded USV, including the respective location of the emitted USV. All statistical analyses are based on this data.
(XLSX)

S1 Table The full linear model for the number of songs recorded in different recording nights and context regions. For context region abbreviations see Fig 1.
(DOCX)

S2 Table The minimal adequate linear model with data from nights 3 and 4 pooled. For context region abbreviations see Fig 1.
(DOCX)

S3 Table ANOVA results of model comparisons on the number of songs in different recording nights and context regions. We tested the full model (MOD.1) against one model without the interaction term of nights and context regions (MOD.2) and against one model with nights 3 and 4 pooled (MOD.3).
(DOCX)

S4 Table The full model on the number of songs recorded in different types of encounters at the contact window during the three nights with direct contact. (DOCX)

S5 Table The minimal adequate linear model with face to face and non-face to face encounters pooled. (DOCX)

S6 Table ANOVA results of model comparisons on the number of songs in different encounters at the contact window in the three recording nights with direct social contact. We tested the full model (MOD.1) against one model without the interaction term of nights and types of encounters (MOD.2), another model with nights 3 and 4 pooled (MOD.3), as well as the minimum adequate model with face to face and non-face to face encounters pooled (MOD.4).
(DOCX)

S7 Table Song and syllable parameters in the male bedding region and in the contact corners. Given is the mean ± sd for both context regions.
(DOCX)
