# Supplementary figures and images for: Communication at the Garden Fence – Context Dependent Vocalization in Female House Mice
Source: PLoS One. 2016 Mar 29;11(3):e0152255. doi: 10.1371/journal.pone.0152255 (PMC4811528; doi:10.1371/journal.pone.0152255)

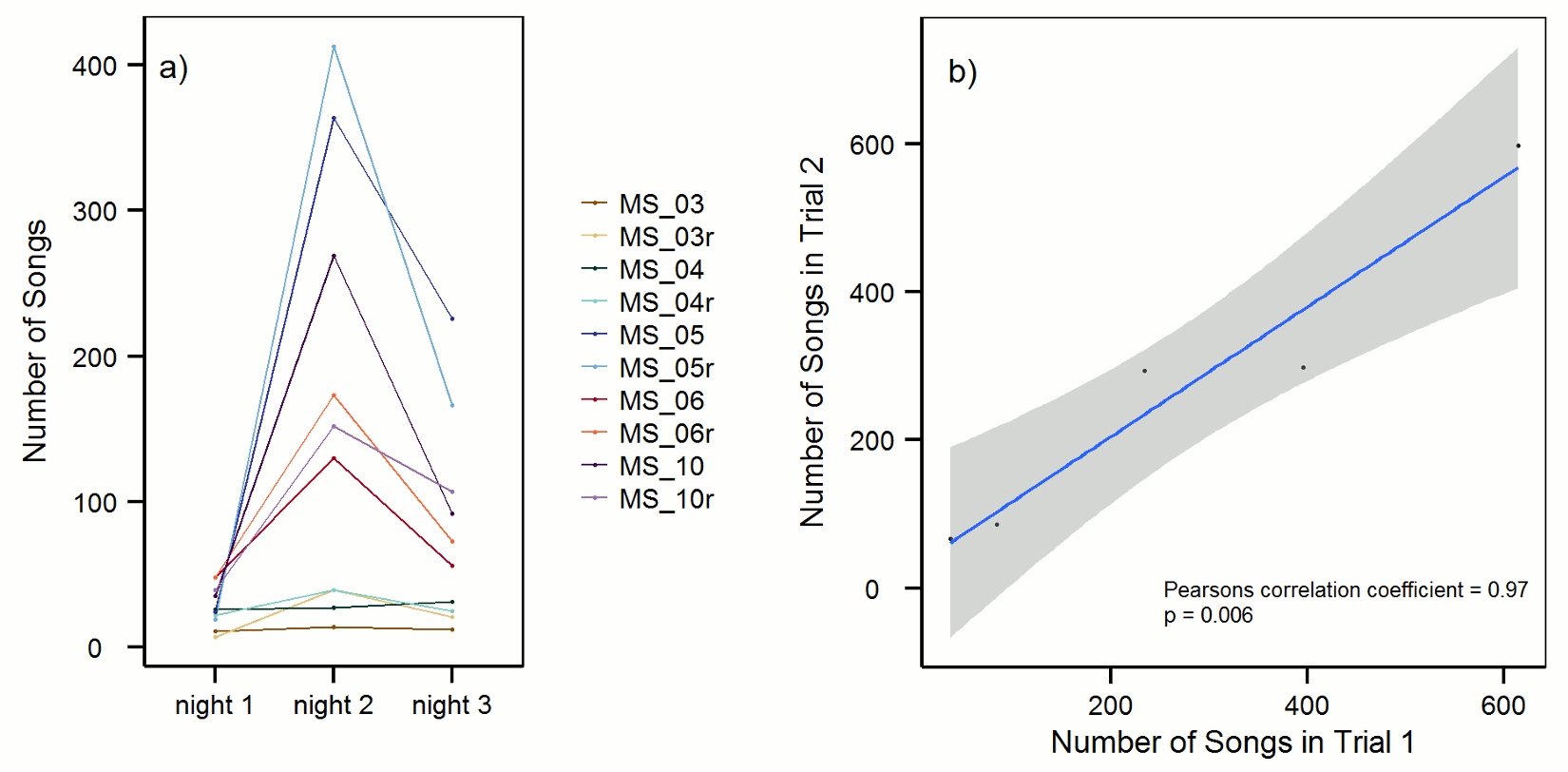

Supplement: S1 Fig — a) The number of songs recorded from each pair of females in the different recording nights, shown for the first and the second trial. The respective repetition for each pair is indicated by an “r” after the pair name. b) The total number of songs recorded from each pair of females in trial 1 and trial 2. (TIFF) [file pone.0152255.s001.tiff]

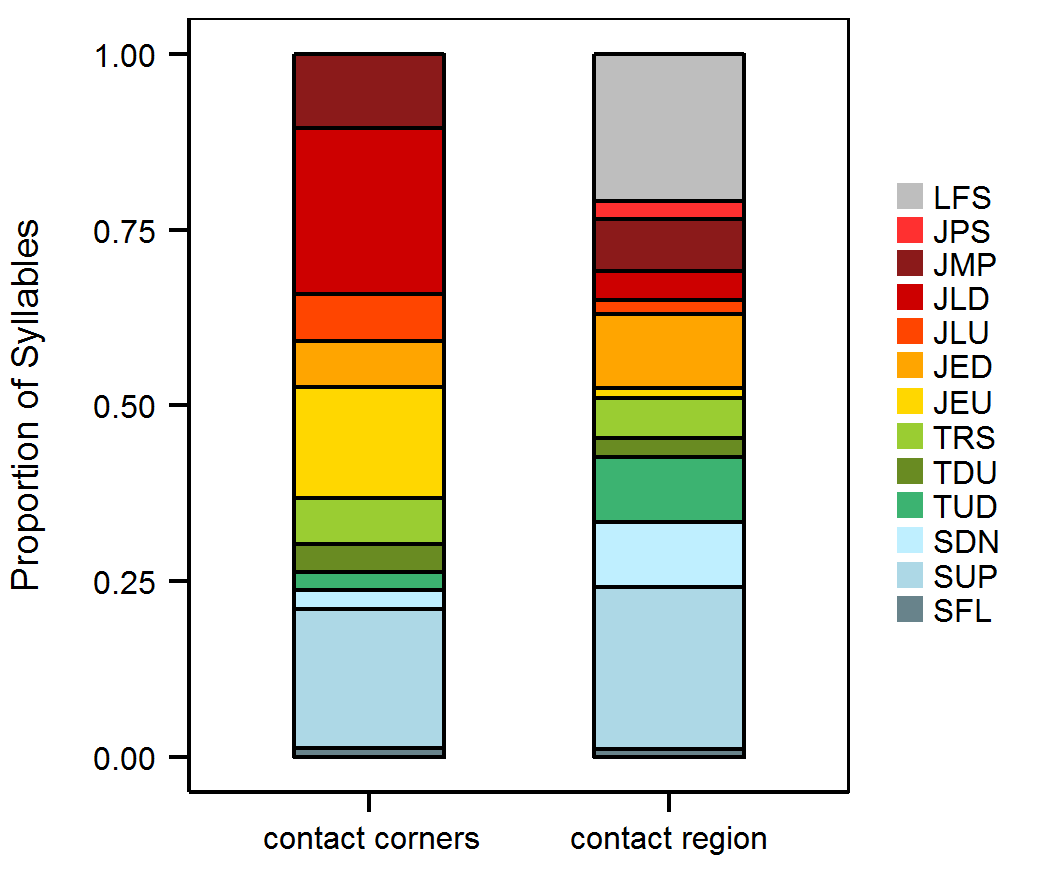

Supplement: S2 Fig — Shown is the proportion of each syllable type in percent. Syllable types are: SFL = Simple-Flat, SUP = Simple-Up, SDN = Simple-Down, TUD = Turn-Up-Down, TDU = Turn- Down-Up, TRS = Turn-Multi, JEU = Jump-Early-Up, JED = Jump-Early-Down, JLU = Jump-Late-Up, JLD = Jump-Late-Down, JMP = Two-Jump, JPS = Jump-Multi, LFS = Low-Frequency-Syllables. For a detailed description of each syllable type see [17]. (TIFF) [file pone.0152255.s002.tiff]
