# Supplementary material for: Communication at the Garden Fence – Context Dependent Vocalization in Female House Mice
Source: PLoS One. 2016 Mar 29;11(3):e0152255. doi: 10.1371/journal.pone.0152255 (PMC4811528; doi:10.1371/journal.pone.0152255)
Supplement: S1 Table — For context region abbreviations see Fig 1. (DOCX) [file pone.0152255.s004.docx]

| **Full model** | | | | |
| --- | --- | --- | --- | --- |
| Formula: Songs ~ Night * ContextRegion + (1 \| Pair) | | | | |
| REML criterion at convergence: 1255.9 | | | | |
| Random effects: | Groups | Name | Variance | Std.Dev. |
|  | Pair |  | 146.3 | 12.1 |
|  | Residual |  | 872.6 | 29.54 |
|  | Number of obs.: | 142 | Groups: | 12 |
| Fixed effects: |  | Estimate | Std. Error | t value |
|  | (Intercept) | 3.917 | 9.215 | 0.425 |
|  | night2 | -2.206 | 12.690 | -0.174 |
|  | night3 | -5.567 | 14.920 | -0.373 |
|  | night4 | -6.270 | 22.990 | -0.273 |
|  | NR | 0.167 | 12.060 | 0.014 |
|  | CC | 11.690 | 12.690 | 0.922 |
|  | CR | 0.000 | 12.060 | 0.000 |
|  | night2:NR | 4.664 | 17.680 | 0.264 |
|  | night3:NR | 2.654 | 20.950 | 0.127 |
|  | night4:NR | 6.383 | 28.490 | 0.224 |
|  | night2:CC | 5.096 | 18.000 | 0.283 |
|  | night3:CC | 6.630 | 19.950 | 0.332 |
|  | night4:CC | 7.953 | 27.560 | 0.289 |
|  | night2:CR | 88.870 | 17.500 | 5.077 |
|  | night3:CR | 38.700 | 19.340 | 2.001 |
|  | night4:CR | 28.480 | 27.250 | 1.045 |
