# Supplementary material for: Communication at the Garden Fence – Context Dependent Vocalization in Female House Mice
Source: PLoS One. 2016 Mar 29;11(3):e0152255. doi: 10.1371/journal.pone.0152255 (PMC4811528; doi:10.1371/journal.pone.0152255)
Supplement: S2 Table — For context region abbreviations see Fig 1. (DOCX) [file pone.0152255.s005.docx]

| **Minimum adequate model with nights 3 and 4 pooled** | | | | |
| --- | --- | --- | --- | --- |
| Formula: Songs ~ newNight^(1)^ + ContextRegion + (1 \| Pair) | | | | |
| REML criterion at convergence: 1286.9 | | | | |
| Random effects: | Groups | Name | Variance | Std.Dev. |
|  | Pair | (Intercept) | 148.7 | 12.19 |
|  | Residual |  | 847.3 | 29.11 |
|  | Number of obs.: | 142 | Groups: | 12 |
| Fixed effects: |  | Estimate | Std. Error | t value |
|  | (Intercept) | 3.917 | 9.111 | 0.430 |
|  | night2 | -2.230 | 12.500 | -0.178 |
|  | nights3+4 | -5.826 | 13.420 | -0.434 |
|  | NR | 0.167 | 11.880 | 0.014 |
|  | CC | 11.670 | 12.500 | 0.933 |
|  | CR | 0.000 | 11.880 | 0.000 |
|  | night2:NR | 4.657 | 17.420 | 0.267 |
|  | nights3+4:NR | 4.116 | 18.310 | 0.225 |
|  | night2:CC | 5.144 | 17.740 | 0.290 |
|  | nights3+4:CC | 7.217 | 17.890 | 0.403 |
|  | night2:CR | 88.900 | 17.250 | 5.154 |
|  | nights3+4:CR | 35.170 | 17.350 | 2.027 |
| (1) nights 3 and 4 pooled | |  |  |  |
