# Supplementary material for: Communication at the Garden Fence – Context Dependent Vocalization in Female House Mice
Source: PLoS One. 2016 Mar 29;11(3):e0152255. doi: 10.1371/journal.pone.0152255 (PMC4811528; doi:10.1371/journal.pone.0152255)
Supplement: S3 Table — We tested the full model (MOD.1) against one model without the interaction term of nights and context regions (MOD.2) and against one model with nights 3 and 4 pooled (MOD.3). (DOCX) [file pone.0152255.s006.docx]

| Models: | MOD.1: Songs ~ Night + ContextRegion + (1 \| Pair) | | | | | | | | |
| --- | --- | --- | --- | --- | --- | --- | --- | --- | --- |
|  | MOD.2: Songs ~ Night * ContextRegion + (1 \| Pair) | | | | | | | | |
|  | MOD.3: Songs ~ newNight^(1)^ * ContextRegion + (1 \| Pair) | | | | | | | | |
|  | Df | AIC | BIC | logLik | deviance | Chisq | Df | Pr(>Chisq) |  |
| MOD.1 | 18 | 1396.6 | 1449.8 | -680.28 | 1360.6 |  |  |  |  |
| MOD.2 | 9 | 1416.3 | 1442.9 | -699.15 | 1398.3 | 0.6221 | 4 | 0.9606 |  |
| MOD.3 | 14 | 1389.2 | 1430.6 | -680.60 | 1361.2 | 37.1051 | 5 | 0.0000 | *** |
| (1) nights 3 and 4 pooled; Signifcance levels: p<0.001 *** | | | | | | | | | |
