# Supplementary material for: Communication at the Garden Fence – Context Dependent Vocalization in Female House Mice
Source: PLoS One. 2016 Mar 29;11(3):e0152255. doi: 10.1371/journal.pone.0152255 (PMC4811528; doi:10.1371/journal.pone.0152255)
Supplement: S4 Table — (DOCX) [file pone.0152255.s007.docx]

| **Full Model** |  |  |  |  |
| --- | --- | --- | --- | --- |
| Formula: Songs ~ Night * Encounter + (1 \| Pair) | | | | |
| REML criterion at convergence: 604.1 | | | | |
| Random effects: | Groups | Name | Variance | Std.Dev. |
|  | Pair |  | 567.2 | 23.82 |
|  | Residual |  | 376.8 | 19.41 |
|  | Number of obs.: | 73 | Groups: | 12 |
| Fixed effects: |  | Estimate | Std. Error | t value |
|  | (Intercept) | 12.204 | 9.3 | 1.312 |
|  | night3 | -14.745 | 10.228 | -1.442 |
|  | night4 | -6.813 | 10.943 | -0.623 |
|  | face to face | 9.542 | 8.612 | 1.108 |
|  | solitary | 47.713 | 8.403 | 5.678 |
|  | night3:face to face | -3.465 | 14.08 | -0.246 |
|  | night4:face to face | -8.942 | 14.996 | -0.596 |
|  | night3:solitary | -20.871 | 13.008 | -1.604 |
|  | night4:solitary | -31.012 | 14.446 | -2.147 |
