# Supplementary material for: Communication at the Garden Fence – Context Dependent Vocalization in Female House Mice
Source: PLoS One. 2016 Mar 29;11(3):e0152255. doi: 10.1371/journal.pone.0152255 (PMC4811528; doi:10.1371/journal.pone.0152255)
Supplement: S5 Table — (DOCX) [file pone.0152255.s008.docx]

| **Minimum adequate model with "face to face” and “non-face to face” encounters pooled** | | | | |
| --- | --- | --- | --- | --- |
| Formula: Songs ~ Night * newEncounter^(1)^ + (1 \| Pair) | | | | |
| REML criterion at convergence: 625.3 | | | | |
| Random effects: | Groups | Name | Variance | Std.Dev. |
|  | Pair |  | 553.3 | 23.52 |
|  | Residual |  | 368.7 | 19.2 |
|  | Number of obs.: | 73 | Groups: | 12 |
| Fixed effects: |  | Estimate | Std. Error | t value |
|  | (Intercept) | 17.285 | 8.003 | 2.16 |
|  | night3 | -16.327 | 6.943 | -2.352 |
|  | night4 | -11.264 | 7.881 | -1.429 |
|  | solitary | 42.631 | 6.976 | 6.111 |
|  | night3:solitary | -19.304 | 10.559 | -1.828 |
|  | night4:solitary | -26.443 | 12.187 | -2.17 |
| (1) face to face and non-face to face encounters pooled. | | | | |
