# Supplementary material for: Communication at the Garden Fence – Context Dependent Vocalization in Female House Mice
Source: PLoS One. 2016 Mar 29;11(3):e0152255. doi: 10.1371/journal.pone.0152255 (PMC4811528; doi:10.1371/journal.pone.0152255)
Supplement: S6 Table — We tested the full model (MOD.1) against one model without the interaction term of nights and types of encounters (MOD.2), another model with nights 3 and 4 pooled (MOD.3), as well as the minimum adequate model with face to face and non-face to face encounters pooled (MOD.4). (DOCX) [file pone.0152255.s009.docx]

| Models: | MOD.1: Songs ~ Night * Encounter + (1 \| Pair) | | | | | | | | |
| --- | --- | --- | --- | --- | --- | --- | --- | --- | --- |
|  | MOD.2: Songs ~ Night + Encounter + (1 \| Pair) | | | | | | | | |
|  | MOD.3: Songs ~ newNight^(1)^ + Encounter + (1 \| Pair) | | | | | | | | |
|  | MOD.4: Songs ~ Night * new Encounter ^(2)^ + (1 \| Pair) | | | | | | | | |
|  | Df | AIC | BIC | logLik | deviance | Chisq | Df | Pr(>Chisq) |  |
| MOD.1 | 11 | 679.81 | 705 | -328.9 | 657.81 | 1.7466 | 3 | 0.6266 |  |
| MOD.2 | 7 | 678.46 | 694.49 | -332.23 | 664.46 |  |  |  |  |
| MOD.3 | 11 | 679.81 | 705 | -328.9 | 657.81 | 0 | 0 | 1 |  |
| MOD.4 | 8 | 675.56 | 693.88 | -329.78 | 659.56 | 4.9036 | 1 | 0.0268 | * |
| (1) nights 3 and 4 pooled; (2) face to face and non-face to face encounters pooled; Signifcance levels: p< 0.05 * | | | | | | | | | |
