# Supplementary material for: Communication at the Garden Fence – Context Dependent Vocalization in Female House Mice
Source: PLoS One. 2016 Mar 29;11(3):e0152255. doi: 10.1371/journal.pone.0152255 (PMC4811528; doi:10.1371/journal.pone.0152255)
Supplement: S7 Table — Given is the mean ± sd for both context regions. (DOCX) [file pone.0152255.s010.docx]

| **Parameter** | **male bedding** | **contact corners** |
| --- | --- | --- |
| soDur | 475.28 ± 489.55 | 500.88 ± 493.89 |
| # of syls | 4.21 ± 2.86 | 4.70 ± 3.60 |
| sylDur | 49.16 ± 36.49 | 43.45 ± 34.66 |
| staFreq | 87.09 ± 23.35 | 77.34 ± 17.06 |
| Slope | -0.09 ± 1.02 | 0.04 ± 0.77 |
| minFreq | 62.80 ± 17.90 | 63.83 ± 14.76 |
| freqBand | 35.02 ± 20.41 | 25.8 ± 17.60 |
| freqCOG | 73.52 ± 18.11 | 73.71 ± 14.59 |
| Jumps | 1.03 ± 0.93 | 0.77 ± 1.00 |
| Turns | 2.32 ± 1.75 | 2.02 ± 2.22 |
